# Downregulation of Matriptase Inhibits Porphyromonas gingivalis Lipopolysaccharide-Induced Matrix Metalloproteinase-1 and Proinflammatory Cytokines by Suppressing the TLR4/NF-*κ*B Signaling Pathways in Human Gingival Fibroblasts

**DOI:** 10.1155/2022/3865844

**Published:** 2022-10-04

**Authors:** Jeong-Mi Kim, Eun-Mi Noh, Yong-Ouk You, Min Seuk Kim, Young-Rae Lee

**Affiliations:** ^1^Department of Biochemistry, Jeonbuk National University Medical School, Jeonju-si, Jeollabuk-do 54896, Republic of Korea; ^2^Department of Oral Biochemistry, And Institute of Biomaterials-Implant, College of Dentistry, Wonkwang University, Iksan City, Jeonbuk 54538, Republic of Korea; ^3^Department of Oral Physiology, And Institute of Biomaterial-Implant, College of Dentistry, Wonkwang University, Iksan City, Jeonbuk 54538, Republic of Korea

## Abstract

Matriptases are cell surface proteolytic enzymes belonging to the type II transmembrane serine protease family that mediate inflammatory skin disorders and cancer progression. Matriptases may affect the development of periodontitis via protease-activated receptor-2 activity. However, the cellular mechanism by which matriptases are involved in periodontitis is unknown. In this study, we examined the antiperiodontitis effects of matriptase on *Porphyromonas gingivalis*-derived lipopolysaccharide (PG-LPS)-stimulated human gingival fibroblasts (HGFs). Matriptase small interfering RNA-transfected HGFs were treated with PG-LPS. The mRNA and protein levels of proinflammatory cytokines and matrix metalloproteinase 1 (MMP-1) were evaluated using the quantitative real-time polymerase chain reaction (qRT-PCR) and an enzyme-linked immunosorbent assay (ELISA), respectively. Western blot analyses were performed to measure the levels of Toll-like receptor 4 (TLR4)/interleukin-1 (IL-1) receptor-associated kinase (IRAK)/transforming growth factor *β*-activated kinase 1 (TAK1), p65, and p50 in PG-LPS-stimulated HGFs. Matriptase downregulation inhibited LPS-induced proinflammatory cytokine expression, including the expression of IL-6, IL-8, tumor necrosis factor-*α* (TNF-*α*), and IL-I*β*. Moreover, matriptase downregulation inhibited PG-LPS-stimulated MMP-1 expression. Additionally, we confirmed that the mechanism underlying the effects of matriptase downregulation involves the suppression of PG-LPS-induced IRAK1/TAK1 and NF-*κ*B. These results suggest that downregulation of matriptase PG-LPS-induced MMP-1 and proinflammatory cytokine expression via TLR4-mediated IRAK1/TAK1 and NF-*κ*B signaling pathways in HGFs.

## 1. Introduction

Periodontal disease is a serious inflammatory disease affecting the gingiva that can lead to tooth damage and other health complications. Periodontal disease is mainly caused by infection with *Porphyromonas gingivalis* (*P. gingivalis*) and is the primary cause of tooth loss owing to damaged soft tissues and bone resorption around the teeth [1, 2]. *P. gingivalis* produces a lipopolysaccharide (PG-LPS) that represents the major virulence factor [3]. LPS produces various proinflammatory cytokines and mediators of inflammation such as interleukin- (IL-) 6, IL-8, tumor necrosis factor-*α* (TNF-*α*), IL-1*β*, prostaglandins, nitric oxide, and matrix metalloproteinases (MMPs) [4, 5]. The underlying mechanism of the inflammatory response induced by PG-LPS relies on its interaction with Toll-like receptor-4 (TLR4). Additionally, PG-LPS upregulates MMPs and cytokines via various signaling pathways by binding to TLR4 [6–8]. Therefore, inhibiting signaling pathways associated with MMPs and cytokine expression may suppress periodontal disease [9].

Human gingival fibroblasts (HGFs) play a major role in periodontal disease mediated by periodontal tissue destruction and inflammation [10–12]. Stimulation of gingival fibroblasts by PG-LPS activates multiple TRL4-mediated signaling pathways [13–15]. The binding of LPS to TLR4 leads to recruitment of myeloid differentiation primary response 88 (MyD88) and formation of complexes with IL-1 receptor-associated kinase which, in turn, leads to the activation of transforming growth factor *β*-activated kinase 1 (TAK1) [16, 17]. Subsequently, this signal transduction process activates I*κ*B kinase (IKK) that induces the expression of MMPs and proinflammatory cytokines via nuclear factor kappa B (NF-*κ*B) activation [18, 19].

MMPs are a family of extracellular matrix- (ECM-) degrading enzymes and play important roles in several disorders including periodontitis, inflammation, tumor invasion, and skin aging [5, 20]. Cells in inflamed periodontal tissues, such as fibroblasts, epithelial cells, and inflammatory cells, have increased expression of various MMPs [21–23]. Among these MMPs, MMP-1 (collagenase I) and MMP-3 (stromelysin) play major roles in periodontal disease development [21, 24, 25]. For example, MMP-1 plays a role in degrading collagen in periodontal tissues during the progression of periodontal disease [26].

Matriptases, are among the most well-studied members of the type II transmembrane serine protease family and are broadly expressed in epithelial compartments of various tissues [27]. Protease-activated receptor-2 (PAR-2), a G protein-coupled receptor, mediates multiple intracellular signaling pathways following proteolytic cleavage at its activation site by trypsin-like serine proteases such as matriptases [28–30]. Recent studies showed that matriptase activity is modulated by intracellular signaling pathways via activation of PAR-2 [30–32]. Activation of PAR-2 plays an important role in the pathogenesis of periodontal diseases caused by *P. gingivalis* [33, 34]. However, the cellular mechanisms by which matriptase mediates periodontitis are not well-known. In the present work, we investigated the inhibitory effect and signaling pathways of matriptase on PG-LPS-induced MMP-1 and proinflammatory cytokine expression in HGFs.

## 2. Materials and Methods

### 2.1. Chemicals and Reagents

PG-LPS was obtained from InvivoGen (San Diego, CA, USA). PG-LPS was solubilized in endotoxin-free water. Dimethyl sulfoxide (cat. no. 472301) was obtained from Sigma-Aldrich (St. Louis, MO, USA). Axon Medchem (Groningen, the Netherlands) provided the PAR-2 antagonist, GB83. M62182 (a TLR4 inhibitor) was obtained from Tocris Bioscience (Abingdon, United Kingdom).

### 2.2. Cell Culture

Primary HGFs were purchased from Science Cell Research Laboratories (cat. #2620; Carlsbad, CA, USA). HGFs were cultured in Dulbecco's modified Eagle's medium supplemented with 10% fetal bovine serum (cat. 16000-044; Thermo Fisher Scientific, Waltham, MA, USA) and a 1% antibiotic solution (antibiotic-antimycotic, 100X; Thermo Fisher Scientific). Cultures were maintained at 37°C in a humidified incubator with 5% CO_2_.

### 2.3. RNA Interference

HGFs were transfected with 100 pmol matriptase-specific small interfering RNA (siRNA) or negative control siRNA (Shanghai Genepharm, Shanghai, China). The human siRNA sequences were as follows: matriptase siRNA, 5′-GUGUCCAGAAGGUCUUCAATT-3′ (sense) and 5′-UUGAAGACCUUCUGGACACTT-3′ (antisense); negative control siRNA, 5′-UUCUCCGAACGUGUCACGUTT-3′ (sense) and 5′-ACGUGACACGUUCGGAGAATT-3′ (antisense). Briefly, HGFs were transfected with 100 pmol siRNA using Lipofectamine® RNAiMAX Transfection Reagent (Invitrogen, Carlsbad, CA, USA) according to the manufacturer's protocol.

### 2.4. Western Blotting Analysis

HGFs were lysed using ice-cold radioimmunoprecipitation assay buffer (Thermo Scientific, Rockford, IL, USA). Cellular proteins (20 *μ*g) were analyzed by sodium dodecyl sulfate-polyacrylamide gel electrophoresis and transferred to Hybond™ polyvinylidene fluoride membranes (GE Healthcare Life Sciences, Buckinghamshire, United Kingdom). Each membrane was blocked with a blocking buffer (5% bovine serum albumin or 5% skim milk) for 2 h at 4°C and then incubated with primary antibodies (1 : 2500 dilution) overnight at 4°C.

Antibodies against IL-1 receptor-associated kinase (IRAK1), TAK1, IKK*α*, and IKK*β*, and the phosphorylated forms of the inhibitory subunit of NF-*κ*B*α* (I*κ*B*α*), I*κ*B kinase *αβ* (IKK*αβ*), IRAK1, and TAK1 were purchased from Cell Signaling Technology (Beverly, MA, USA). Antibodies specific for proliferating cell nuclear antigen and horseradish peroxidase-conjugated secondary IgGs were obtained from Santa Cruz Biotechnology (Santa Cruz, CA, USA) and Cell Signaling Technology, respectively. Antibodies specific for matriptase were obtained from R&D Systems (Minneapolis, MN, USA). Antibodies specific for p50 and p65 were obtained from Abcam (Cambridge, UK). The blots were washed in Tris-buffered saline with 0.2% Tween-20 and then incubated with secondary horseradish peroxidase-conjugated goat anti-mouse or anti-rabbit for 1 h at 4°C. The *β*-actin antibody was from Sigma-Aldrich. *β*-Actin and proliferating cell nuclear antigen were used as loading controls. Protein expression levels were determined using a Mini HD6 Image Analyzer and Alliance 1D (UVItec Cambridge; Cleaver Scientific, Rugby, United Kingdom).

### 2.5. RNA Isolation and Quantitative Real-Time Polymerase Chain Reaction (qRT-PCR)

Total RNA was extracted from cells using TRIzol reagent (Invitrogen). Complementary DNA was prepared from 1 *μ*g of total RNA using the PrimeScript™ RT Reagent Kit (Takara Bio, Shiga, Japan) according to the manufacturer's instructions. The RNA expression levels of *MMP-1* and *glyceraldehyde 3-phosphate dehydrogenase* (*gapdh*) were analyzed using the qRT-PCR with the StepOnePlus Real-Time PCR System thermocycler and SYBR® Green PCR master mix reagent (Applied Biosystems, Foster City, CA, USA). The primers used included the following: human *MMP1* (NM 002424.2) (sense: 5′-AGTGACTGGGAAACCGATGCTGA-3′ and antisense: 5′-CTCTTGGCAAATCTGGCCTGTAA-3′); *IL6* (sense: 5′-TACCCCCAGGAGAAGATTCC-3′ and antisense: 5′-GCCATCTTTGG AAGGTTCAG-3′); *IL8* (sense: 5′-AGACAGCAGAGCACACAAGC-3′ and antisense: 5′-ATGGTTCCTTCCGGTGGT-3′); *IL1β* (sense: 5′-TCCTGCGTGTTGAAAGATGATAA-3′ and antisense: 5′-CAAATCGCTTTTCCATCTTCTTC-3′); *TNFα* (sense: 5′-CTGCTGCACTTTGGAGTGAT-3′ and antisense: 5′-AGATGATCTGACTGCCTGGG-3′); *GAPDH* (NM 002046) (sense: 5′-ATGGAAATCCCATCACCATCTT-3′ and antisense: 5′-CGCCCCACTTGATTTTGG-3′). The qRT-PCR cycling conditions were as follows: initial denaturation at 95°C for 10 min, 40 cycles of 95°C for 15 sec and 60°C for 1 min, followed by a melting curve ranging from 95°C for 15 sec, 60°C for 1 min, to 95°C for 15 sec. To control for variations in mRNA concentrations, all results were normalized to the expression level of the GAPDH housekeeping gene. Relative quantitation was performed using the comparative *ΔΔ*Ct method [35].

### 2.6. Enzyme-Linked Immunosorbent Assay (ELISA)

MMP-1 in conditioned medium was quantified using a sandwich ELISA kit (cat. #F1M00, R&D Systems) according to the manufacturer's protocol. HGFs were transfected with matriptase siRNA and stimulated with PG-LPS for 24 and 48 h at 37°C. Standards and sample were added and incubated at room temperature for 3 h. After 4 washes, APMA (p-aminophenylmercuric acetate) was added to the standards and sample wells for 2 h at room temperature. After 4 washes, substrate was added to each well for 17~20 h at 37°C. The reaction was determined (RFU) using a microplate reader (Sunrise™, Tecan Group, Männedorf, Switzerland). MMP-1 activation was given as the active MMP-1 levels (ng/mL) in the cell culture medium. Absorbance at 450 nm was measured using a microplate reader (Sunrise™, Tecan Group, Männedorf, Switzerland).

### 2.7. Analysis of Nuclear and Cytoplasmic Extracts

HGFs were transfected with 100 pmol siRNA against matriptase or negative control siRNA using the RNAiMAX Transfection Reagent (Thermo Fisher Scientific) for 24 h, then incubated with PG-LPS for 3 h. Nuclear and cytoplasmic proteins were extracted using the NE-PER Nuclear and Cytoplasmic Extraction Kit (Pierce, Rockford, IL, USA), according to the manufacturer's instructions. The protein concentrations in the membrane, cytosol, and nuclear fractions were determined using a protein assay.

### 2.8. Statistical Analysis

Data are expressed as means ± standard error. Statistical significance was determined using a one-way analysis of variance followed by the Scheffe post hoc test using Microsoft Excel (Redmond, WA, USA). A value of *p* < 0.05 and *p* < 0.01 represent significant differences. All experiments were performed in triplicate.

## 3. Results

### 3.1. Effect of Matriptase Downregulation on PG-LPS-Induced MMP-1 Expression in HGFs

**Figure 1:**
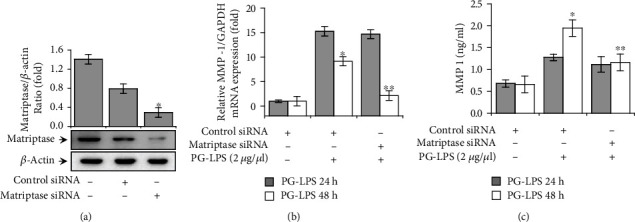
Effect of matriptase on PG-LPS-induced MMP-1 expression in HGFs. (a) HGFs were transfected with matriptase or negative control siRNA for 24 h, and the expression of matriptase was assessed via western blotting. (b) siRNA-transfected HGFs were treated with PG-LPS for 24 and 48 h. MMP-1 mRNA levels were analyzed via the real-time polymerase chain reaction using GAPDH as an internal control. (c) MMP-1 protein levels in HGFs were detected using an enzyme-linked immunosorbent assay. Values are presented as means ± standard error of the mean of three independent experiments. ^∗^*p* < 0.05 vs. untreated control; ^∗∗^*p* < 0.01 vs. PG-LPS. PG-LPS: *Porphyromonas gingivalis*-derived lipopolysaccharide; MMP-1: matrix metalloproteinase 1; HGFs: human gingival fibroblasts; siRNA: small interfering RNA; GAPDH: glyceraldehyde 3-phosphate dehydrogenase.

To investigate the effect of matriptase on PG-LPS-induced MMP-1 expression, we first confirmed that matriptase expression was downregulated by approximately 2.7-fold compared to control siRNA after transfection with matriptase siRNA (Figure 1(a)). The *MMP1* mRNA expression in HGFs was analyzed by the qRT-PCR. Downregulation of matriptase expression decreased PG-LPS-induced mRNA expression of *MMP1* by approximately 4.3-fold at 48 h compared to control siRNA (Figure 1(b)). The ELISA confirmed that PG-LPS treatment of HGFs resulted in an increase in MMP-1 protein secretion, while matriptase siRNA significantly diminished this increase by approximately 1.7-fold at 48 h compared to control siRNA (Figure 1(c)). These results indicate that matriptase involved in PG-LPS-mediated MMP-1 expression.

### 3.2. Effect of Matriptase Downregulation on the Expression of PG-LPS-Induced Proinflammatory Cytokines in HGFs

**Figure 2:**
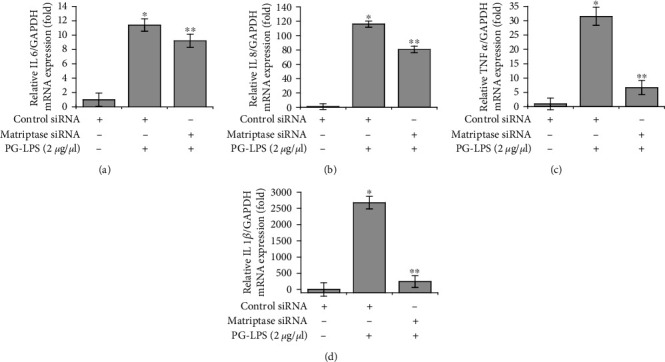
Effect of matriptase on PG-LPS-induced proinflammatory cytokine expression in HGFs. (a–d) HGFs were transfected with negative control or matriptase small interfering RNA for 24 h, then with PG-LPS for 6 h. Total cellular mRNA was extracted and the mRNA levels of *IL6*, *IL8*, *TNFα*, and *IL1β* were determined via the quantitative real-time polymerase chain reaction. Values are presented as means ± standard error of the mean of three independent experiments. ^∗^*p* < 0.05 vs. untreated control; ^∗∗^*p* < 0.01 vs. PG-LPS. PG-LPS: *Porphyromonas gingivalis*-derived lipopolysaccharide; HGFs: human gingival fibroblasts; *IL6*: *interleukin6*; *IL8*: *interleukin8*; *TNFα*: *tumor necrosis factor α*; *IL1β*: *interleukin 1β*.

Previous studies have suggested a pivotal role of proinflammatory cytokines in periodontal diseases [25, 36]. Therefore, we evaluated the effects of matriptase downregulation on proinflammatory cytokine expression in HGFs. Matriptase siRNA-transfected HGFs were exposed to PG-LPS for 6 h and then the mRNA was extracted and analyzed. PG-LPS stimulation dramatically increased mRNA expression levels by approximately 11.4-fold for *IL6*, 116.1-fold for *IL8*, 31.5-fold for *TNFα*, and 2679.1-fold for *IL1β* compared to control siRNA, whereas matriptase siRNA-transfected HGFs showed considerably decreased by approximately 9.2-fold for *IL6*, 80.7-fold for *IL8*, 6.7-fold for *TNFα*, and 242.8-fold for *IL1β* (Figure 2). These results indicate that matriptase involved in PG-LPS-induced proinflammatory cytokine expression.

### 3.3. Downregulation of Matriptase Inhibited PG-LPS-Induced IRAK1/TAK1/NF-*κ*B Activation in HGFs

**Figure 3:**
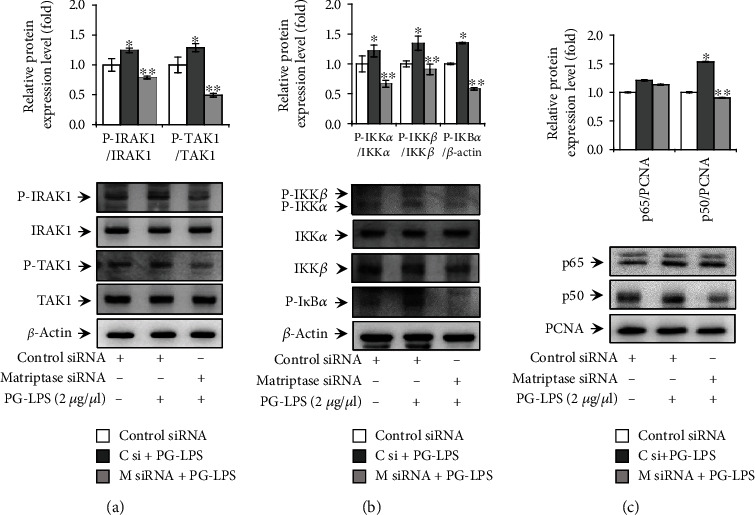
Effect of matriptase on PG-LPS-induced IRAK1 and NF-*κ*B activation in HGFs. (a) HGFs were transfected with negative control or matriptase small interfering RNA for 24 h. Transfected cells were treated with PG-LPS for 5 min, and cell lysates were prepared for western blotting. The levels of total and phosphorylated IRAK1 and TAK1 are shown. (b) Transfected HGFs were treated with PG-LPS for 3 h, after which cytoplasmic and nuclear extracts were obtained. Western blotting was performed to analyze levels of the upstream signaling molecules of NF-*κ*B in the cytoplasmic extracts. (c) The blot was probed with an anti-*β*-actin antibody to assess equal loading, and the nuclear levels of NF-*κ*B (p65 and p50) were determined. The blot was reprobed with an antibody against proliferating cell nuclear antigen to confirm equal loading. Values are presented as means ± standard error of the mean of three independent experiments. ^∗^*p* < 0.05 vs. untreated control; ^∗∗^*p* < 0.01 vs. PG-LPS. PG-LPS: *Porphyromonas gingivalis*-derived lipopolysaccharide; HGFs: human gingival fibroblasts; IRAK1: interleukin-1 receptor-associated kinase 1; TAK1: transforming growth factor *β*-activated kinase 1; NF-*κ*B: nuclear factor kappa-light-chain-enhancer of activated B cells.

The binding of LPS to TLR4 increases MMP and proinflammatory cytokine expression via various signaling pathways such as IRAK, TAK, and NF-*κ*B during the development of periodontitis [13, 16, 37]. Therefore, to investigate the effects of matriptase downregulation on LSP-PG-induced IRAK1, TAK1, and NF-*κ*B activation, we transfected HGFs with matriptase siRNA. Matriptase siRNA reduced the phosphorylation of IRAK1 and TAK1 at 5 min after PG-LPS treatment (Figure 3(a)). Additionally, matriptase siRNA transfection suppressed the phosphorylation of IKK*αβ* and I*κ*B*α* in the cytoplasmic fraction and reduced the nuclear translocation of NF-*κ*B p50 (Figures 3(b) and 3(c)). These results indicate that matriptase was involved in PG-LPS-induced TLR4 signaling-mediated MMP-1 and proinflammatory cytokine expression in HGFs.

### 3.4. Effect of a TLR4 Inhibitor on PG-LPS-Induced IRAK1/TAK1/NF-*κ*B Activation and Proinflammatory Cytokine Expression in HGFs

**Figure 4:**
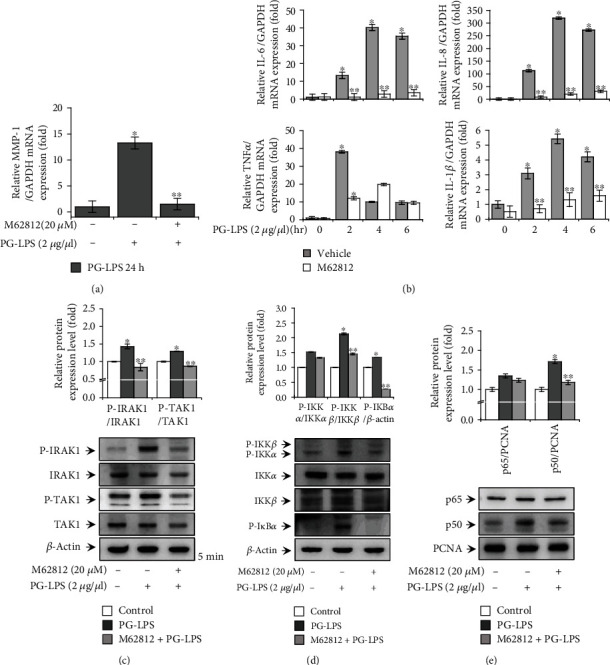
Effect of the TLR4 inhibitor, M62812, on PG-LPS-induced IRAK1/NF-*κ*B activation in HGFs. (a) Cells were pretreated with M62812 for 1 h and then stimulated with PG-LPS for 24 h. MMP-1 mRNA levels were analyzed via the quantitative real-time polymerase chain reaction (qRT-PCR) using GAPDH as an internal control. (b) Cells were pretreated with M62812 for 1 h and then stimulated with PG-LPS for 0, 2, 4, and 6 h. Total cellular mRNA was extracted, and the mRNA levels of *IL6*, *IL8*, *TNFα*, and *IL1β* were determined via the qRT-PCR. (c) Cells were pretreated with M62812 for 1 h and then stimulated with PG-LPS for 5 min. Cell lysates were analyzed via western blotting to determine the levels of total and phosphorylated IRAK1 and TAK1 proteins. (d) Cells were pretreated with M62812 and then stimulated with PG-LPS for 3 h, after which nuclear and cytoplasmic extracts were prepared. Cytoplasmic extracts were used to analyze the upstream signaling molecules of NF-*κ*B, p-I*κ*B*α*, p-IKK*αβ*, IKK*α*, and IKK*β* via western blotting. (e) The blot was probed with an anti-*β*-actin antibody to assess equal loading, and the nuclear levels of NF-*κ*B (p65 and p50) were determined. The blot was reprobed with an antibody against proliferating cell nuclear antigen to confirm equal loading. Values are presented as means ± standard error of mean of three independent experiments. ^∗^*p* < 0.05 vs. untreated control; ^∗∗^*p* < 0.01 vs. PG-LPS. PG-LPS: *Porphyromonas gingivalis*-derived lipopolysaccharide; HGFs: human gingival fibroblasts; IRAK1: interleukin-1 receptor-associated kinase 1; TAK1: transforming growth factor *β*-activated kinase 1; TLR4: toll-like receptor 4; NF-*κ*B: nuclear factor kappa-light-chain-enhancer of activated B cells; IKK: I*κ*B kinase; I*κ*B*α*: inhibitor of NF-*κ*B; *IL6*: *interleukin6*; *IL8*: *interleukin8*; *TNFα*: *tumor necrosis factor α*; *IL1β*: *interleukin 1β*.

The TLR4 pathway plays a major role in the progression of periodontal diseases. First, to investigate the connection of matriptase on PG-LPS-induced TLR4 expression, we confirmed that TLR4 expression was regulated by transfection with matriptase siRNA. PG-LPS treatment of resulted in an increase in TLR4 expression at 24 h, whereas matriptase siRNA diminished this increase (supplementary Figure 1). Thus, these results indicate that matriptase was involved in PG-LPS-mediated TLR4 expression. Next, to elucidate the role of the TLR4 signaling pathway in the response of HGFs to PG-LPS, we treated these cells with M62812, a TLR4 inhibitor. We then determined the phosphorylation or expression of certain key components of the TLR4 pathway including IRAK, TAK1, IKK, I*κ*B*α*, and NF-*κ*B (subunits p65 and p50). M62812 treatment reduced p-IRAK, p-TAK1, p-IKK*αβ*, and p-I*κ*B*α* levels in the cytoplasmic fraction and nuclear translocation of NF-*κ*B p50 (Figures 4(c)–4(e)). Additionally, we found that PG-LPS stimulation dramatically increased mRNA expression levels by approximately 13.3-fold for *MMP1* at 24 h, 40.2-fold for *IL6* at 4 h, 319.5-fold for *IL8* at 4 h, 38.2-fold for *TNFα* at 2 h, and 5.4-fold for *IL1β* at 4 h compared to control siRNA, whereas M62812 treatment showed considerably decreased by approximately 1.5-fold for *MMP1*, 2.8-fold for *IL6*, 20.5-fold for *IL8*, 11.9-fold for *TNFα*, and 1.3-fold for *IL1β* (Figures 4(a) and 4(b)). These findings suggest that PG-LPS stimulation regulates MMP-1 and proinflammatory cytokine expression via TRL4-mediated IRAK1/TAK1 and NF-*κ*B activation in HGFs.

### 3.5. Inhibition of PAR-2 Suppressed PG-LPS-Induced MMP-1 Expression, IRAK1/TAK1 Activation, and Proinflammatory Cytokine Expression in HGFs

**Figure 5:**
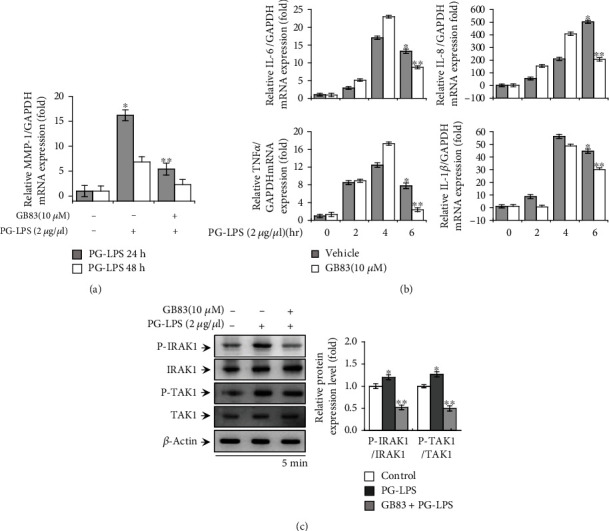
Effect of the PAR-2 inhibitor, GB83, on PG-LPS-induced MMP expression, IRAK1/TAK1 activation, and proinflammatory cytokine expression in HGFs. (a) Cells were pretreated with GB83 for 1 h and then stimulated with PG-LPS for 24 and 48 h. *MMP1* mRNA levels were analyzed via the quantitative real-time polymerase chain reaction (qRT-PCR) using GAPDH as an internal control. (b) Cells were pretreated with GB83 for 1 h and then stimulated with PG-LPS for 0, 2, 4, and 6 h. Total cellular mRNA was extracted, and the mRNA levels of *IL6*, *IL8*, *TNFα*, and *IL1β* were determined via the qRT-PCR. (c) Cells were pretreated with GB83 for 1 h and then stimulated with PG-LPS for 5 min. Cell lysates were subjected to western blotting to determine the levels of total and phosphorylated IRAK1 and TAK1 proteins. Values are presented as means ± standard error of mean of three independent experiments. ^∗^*p* < 0.05 vs. untreated control; ^∗∗^*p* < 0.01 vs. PG-LPS. MMP: matrix metalloproteinase; PG-LPS, *Porphyromonas gingivalis*-derived lipopolysaccharide; HGFs: human gingival fibroblasts; IRAK1: interleukin-1 receptor-associated kinase 1; TAK1: transforming growth factor *β*-activated kinase 1; *IL6*: *interleukin6*; *IL8*: *interleukin8*; *TNFα*: *tumor necrosis factor α*; *IL1β*: *interleukin 1β*; GAPDH: glyceraldehyde 3-phosphate dehydrogenase.

Matriptase activity is regulated by intracellular signaling via PAR-2 activation [29–31]. Therefore, to evaluate the role of PAR-2 in the response of HGFs to treatment with PG-LPS, we determined the effect of a PAR-2 inhibitor, GB83, on *MMP1*, proinflammatory cytokine expression, and IRAK1/TAK1 activation. PG-LPS stimulation dramatically increased mRNA expression levels by approximately 16.2-fold for *MMP1* at 24 h, 13.3-fold for *IL6* at 6 h, 503.2-fold for *IL8* at 6 h, 7.8-fold for *TNFα* at 6 h, and 44.6-fold for *IL1β* at 6 h compared to control siRNA, whereas GB83 treatment showed considerably decreased by approximately 6.8-fold for *MMP1*, 8.7-fold for *IL6*, 206.5-fold for *IL8*, 2.4-fold for *TNFα*, and 30.0-fold for *IL1β* (Figures 5(a) and 5(b)). The treatment additionally attenuated PG-LPS-mediated IRAK1/TAK1 signaling (Figure 5(c)). These results indicate that PAR-2 activity was involved in PG-LPS-induced MMP-1 and proinflammatory cytokine expression in HGFs.

## 4. Discussion

Previous studies suggested that matriptase plays an important role in the pathogenesis of periodontal disease caused by *P. gingivalis*. However, the cellular mechanisms via which matriptase mediates periodontal disease have not been studied in detail. Our results demonstrated the role of matriptase on PG-LPS-induced MMP-1 and inflammatory cytokine expression in HGFs. Specifically, matriptase knockdown considerably suppressed the PAR-2-mediated TLR4 signaling pathway that was activated by PG-LPS. Our study is the first to show that matriptase is involved in TLR4 signaling by PG-LPS.

Periodontal disease is the most common inflammatory disease and one of the main causes is *P. gingivalis* infection [38, 39]. LPS, a major component of the outer membrane in *P. gingivalis*, binds to one of the TLRs, TLR4, which is one of the main virulence factors that induce inflammation [18, 40]. The stimulation of HGFs by PG-LPS activates multiple TRL4-mediated signaling pathways [15, 18, 41]. The binding of LPS to TLR4 results in the recruitment of MyD88, which forms a complex with IRAK and another TIR-containing adapter molecule, Mal (similar to the MyD88 adapter), followed by the activation of TAK1 [15–17, 42]. This activation leads to activation of the IKK/NF-*κ*B pathways [42, 43]. In particular, IKK activation induces the expression of MMP and proinflammatory cytokines via phosphorylation of I*κ*B*α* and translocation of NF-*κ*B subunits to the nucleus [18, 44]. These proinflammatory cytokines and MMP can induce periodontal tissue injury [21, 22, 45]. To clarify the signaling mechanism of TLR4, we demonstrated the inhibitory effect of a TLR4 inhibitor (M62812) on PG-LPS-induced MMP-1 and proinflammatory cytokine expression in HGFs (Figure 4).

Previous studies have indicated that MMPs play major roles in the degradation of the bone matrix and ECM in periodontal disease development [46, 47]. In active periodontal disease, the progressive destruction of periodontal tissue and alveolar bone is increased through the expression of MMPs and inflammatory cytokines [45, 47]. Various MMPs, such as MMPs-1, 2, 3, 8, 9, and 13, are involved in the loss of periodontal tissue [21, 22, 48]. Among these MMPs, MMP-1 is the main component of the periodontal tissue matrix and plays a major role in periodontal ECM degradation and remodeling [21, 48, 49].

In periodontal diseases, proinflammatory cytokine activity is regulated by bacteria and their products [50–52]. IL-6 plays an important role in continuous tissue destruction and bone resorption via osteoclast differentiation in the infected periodontal site [52]. IL-8 induces neutrophil migration to periodontal lesions. This can weaken periodontal tissues by generating intracellular reactive oxygen species and increasing MMP expression and the release of lysosomal enzymes [53, 54].

During periodontitis pathogenesis, IL-1 is involved in the inflammatory response and ECM remodeling through increased expression of various factors including cytokines, MMPs, reactive oxygen species, nitric oxide synthase, and prostaglandins [55–57]. Moreover, it is well-known that stimulation of HGFs with IL-1*β* produces IL-6, IL-8, and TNF-*α* [58, 59]. TNF-*α* is secreted primarily by immune cells or fibroblasts, and it induces the production of MMPs, cytokines, prostaglandin E2, cell adhesion molecules, and factors involved in bone resorption [60, 61]. Therefore, because the regulation of MMP-1 and proinflammatory cytokines is important for the treatment of periodontitis, we demonstrated that downregulating matriptase inhibited the PG-LPS-induced MMP-1 and proinflammatory cytokine expression in HGFs (Figures 1 and 2).

Matriptases are among the most well-studied members of the type II transmembrane serine protease family and are broadly expressed in epithelial compartments of various tissues [27, 62, 63]. A reduction of matriptase activity has been implicated in cell homeostasis, inflammation, osteoarthritis, oral epithelium, and epithelial carcinomas such as breast, colon, prostate, ovary, uterus, cervix, and skin cancer [64–69].

Currently, existing matriptase inhibitors include monoclonal antibodies, peptide-based inhibitors, and small molecule inhibitors, and these have been mainly developed as anticancer drugs. However, the current development of chemical and biochemical matriptase inhibitors is insufficient. The aim of this study was to improve the development of agents that suppress periodontal disease through an enhanced understanding of how to regulate various critical mechanisms. Matriptase regulates multiple intracellular signaling pathways by cleaving the activation site of PAR-2, a G protein-coupled receptor [28–30]. In previous studies, PAR-2 was implicated in the pathogenesis of periodontal disease caused by *P. gingivalis* [33, 34]. Furthermore, it has been reported that signaling via the interaction of PAR-2 and TLR4 induces MMP and cytokine expression [70–72]. Nevertheless, the cellular mechanism via which matriptase regulates the MMPs and proinflammatory cytokines involved in periodontitis was unknown. Therefore, we confirmed the effect of a PAR-2 inhibitor (GB83) on the signaling pathway of PG-LPS-induced MMP-1 and proinflammatory cytokine expression in HGFs (Figure 5). Additionally, we confirmed the inhibitory effect on the signaling pathway of PG-LPS-induced MMP-1 and proinflammatory cytokine expression via the downregulation of matriptase in HGFs (Figure 3).

## 5. Conclusions

Our study demonstrated the signal transduction mechanism by which matriptase affects PG-LPS-induced expression of MMP-1 and proinflammatory cytokines in HGFs. Our data showed that decreasing matriptase inhibited PG-LPS-induced MMP-1 and proinflammatory cytokine expression via TLR4-mediated IRAK1/TAK1 and NF-*κ*B signaling pathways in HGFs. These results provide insights into the therapeutic potential of matriptase for preventing and treating periodontal disease.

## Data Availability

The data used to support the findings of this study are included within the article.

## Supplementary Materials

Supplementary Materials Supplementary 1. Supplement Figure 1: protein level of TLR4 in response to matriptase siRNA.
