# Supplementary material for: Downregulation of Matriptase Inhibits Porphyromonas gingivalis Lipopolysaccharide-Induced Matrix Metalloproteinase-1 and Proinflammatory Cytokines by Suppressing the TLR4/NF-κB Signaling Pathways in Human Gingival Fibroblasts
Source: Biomed Res Int. 2022 Oct 4;2022:3865844. doi: 10.1155/2022/3865844 (PMC9553488; doi:10.1155/2022/3865844)

Supplement Figure1

HGFs were transfected with matriptase of negative control siRNA for 24h and the expression of TLR4 was assessed via western blotting. siRNA-transfected HGFs were treated with PG-LPS for 12, 24 and 48h. The blot was probed with and anti-β-actin antibody to assess equal loading.

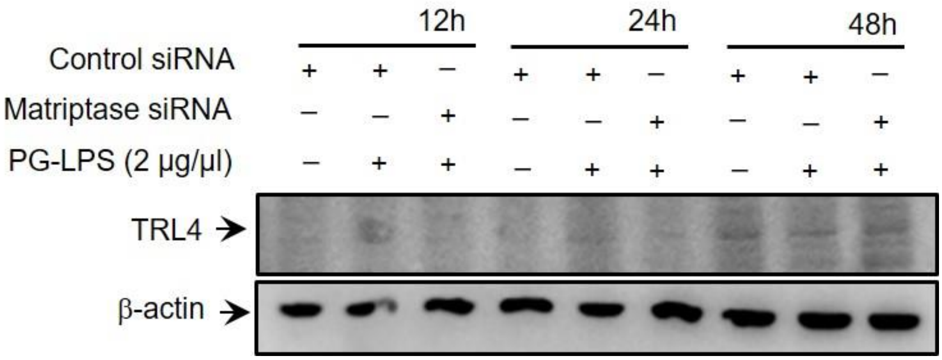

Supplement: Supplementary Materials — Supplementary 1. Supplement Figure 1: protein level of TLR4 in response to matriptase siRNA. [file 3865844.f1.pdf]
